# Mapping the dispersion of water wave channels

**DOI:** 10.1038/s41598-018-21462-2

**Published:** 2018-02-20

**Authors:** David J. Apigo, Alokik Kanwal, John Palmieri, Kyle F. Dobiszewski, Reginald C. Farrow, Gordon A. Thomas, Emil V. Prodan, Camelia Prodan

**Affiliations:** 1 Department of Physics, New Jersey Institute of Technology, Newark, NJ USA; 2 Department of Biomedical Engineering, New Jersey Institute of Technology, Newark, NJ USA; 3 Albert Dorman Honors College, New Jersey Institute of Technology, Newark, NJ USA; 4 Department of Physics and Department of Mathematical Sciences, Yeshiva University, New York, New York USA

## Abstract

Large classes of electronic, photonic, and acoustic crystals and quasi-crystals have been predicted to support topological wave-modes. Some of these modes are stabilized by certain symmetries but others occur as pure wave phenomena, hence they can be observed in many other media that support wave propagation. Surface water-waves are mechanical in nature but very different from the elastic waves, hence they can provide a new platform for studying topological wave-modes. Motivated by this perspective, we report theoretical and experimental characterizations of water-wave crystals obtained by periodic patterning of the water surface. In particular, we demonstrate the band structure of the spectra and existence of spectral gaps.

## Introduction

Wave behavior in photonic, phononic, and acoustic systems has been previously studied theoretically^1–5^ and experimentally^4,6–11^. In a study by Man *et al*. the band structures for a 3-dimensional (3D) quasicrystal (QC) and a 3D diamond structure were acquired by measuring the transmission of microwaves across the system^6^. This novel method provided a photonic way of imaging the Brillouin zone for both lattices and demonstrated properties of a well-behaved system (the diamond structure) and one in which a gap is present (the quasicrystal). In an acoustic topological case, He *et al*.^7^ demonstrate an acoustic topological insulator by measuring the propagation of sound across a system composed of an ordinary phononic crystal and a topological phononic crystal. In separate experiments, Xiao *et al*. and Peng *et al*. demonstrated wave behavior and topological states in periodic acoustic systems. Kraus *et al*. demonstrated photonic wave behavior in QCs^5^. Nash *et al*. performed a study by utilizing a lattice of spinning gyroscopes and demonstrated robust topological properties by measuring the rotations of the gyroscopes due to bursts of air^10^.

This paper reports a platform for quantitatively studying one-dimensional (1D) mechanical waves in fluids. The platform consists of an experimental part designed to map the phonon spectrum and visualising the wave patterns, and a theory that bridges shallow and deep water conditions. For exemplification we use flat-walled channels and periodically patterned channels. The proposed system is significantly different from previously reported photonic and acoustic systems and presents, to our knowledge, for the first time a physical way to map the phonon spectrum of water-wave crystals in various types of channels. Resonant modes can be determined by scanning a wide frequency range and taking images or measuring the amount of light attenuated across the system utilizing laser/photodiode pairs. Unlike Chladni plates, which provide beautiful visualizations of resonant patterns and are useful in measuring modal frequencies in solids^12^, this apparatus provides quantitative data pertaining to fluids.

The flexibility of this system makes it extremely useful for future studies of other types of waveguides, in particular QCs. This paper reports findings on the phonon spectrum of fluids in flat-walled and periodic waveguides; however, this apparatus is not limited to these designs. While here we demonstrate control with periodic paternings, particularly that we can map bulk spectra and open spectral gaps, the platform can be used effectively for studying quasi-periodic or quasi-crystalline patterns displaying topological edge modes.

## Surface water-waves: A theoretical account

The propagation of ripples on the surface of water is a common example of wave propagation. However, determining the speed of this propagation is not as simple as it might seem^13–17^. Indeed, the standard hydrodynamic equations lead to two non-linear boundary equations [^17^, p. 5] that are responsible for the wave phenomena at the surface of a body of water. There are three important length scales in this problem: (1) the amplitude of the wave *A*, (2) the wavelength *λ*, and (3) the water depth *h*. Asymptotic expansions of the non-linear equations can be carried in the limits of small Ursell parameter^15^:

1 $$U=\frac{A}{\lambda }{(\frac{\lambda }{h})}^{3}\ll \mathrm{1,}$$

leading to linearized wave-equations. Different expansions are used^14,15^, depending on the range of values of the parameters *A*/*λ*, *A*/*h* and *λ*/*h*, such as: (1) the shallow water characterized by small *A*/*h*, (2) deep water characterized by small *A*/*h* and small *λ*/*h* and (3) the intermediate regime characterized by small Ursell parameter. A unified treatment of these regimes is provided in^16^.

In the following section we provide a unifying theory of the standing wave-modes in the limit of small amplitudes. It parallels the plasmon hybridization theory^18,19^, where the solutions of the coupled hydro- and electro-dynamic equations are expanded in elementary modes and an effective Lagrangian is constructed for the amplitudes of these modes. Using specific examples, we show that the method reproduces the traditional treatments of water-wave phenomena.

### Theory of standing water-waves

**Figure 1:**
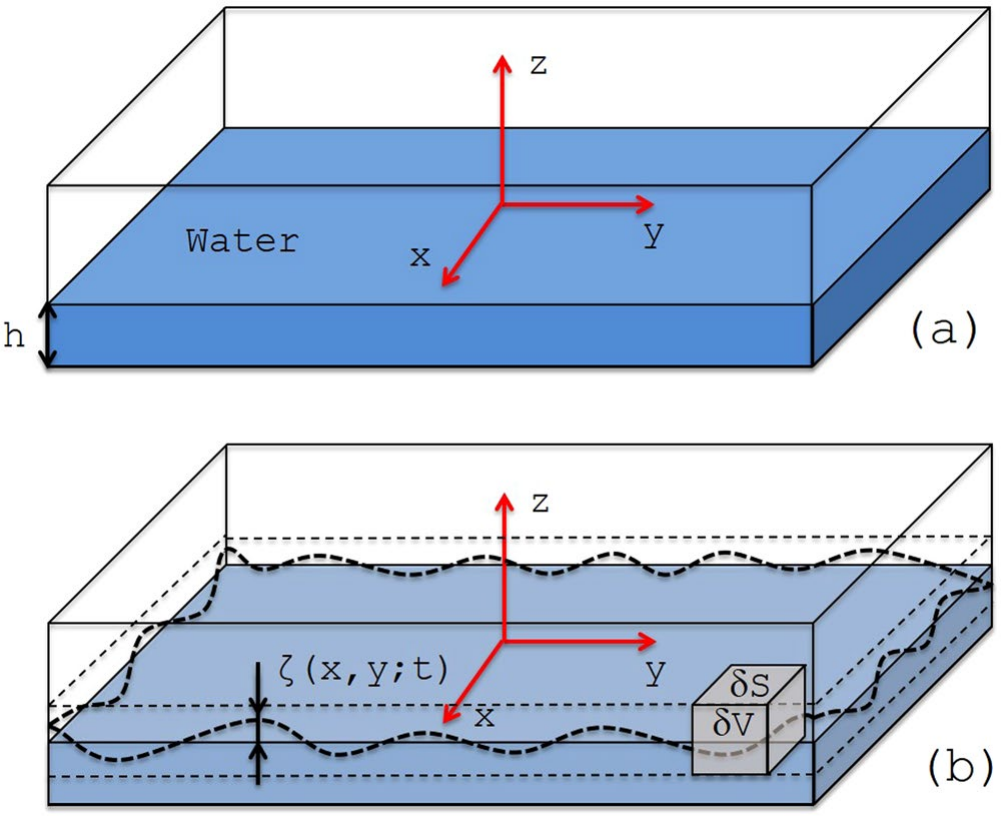
A rectangular water tank with water (**a**) at equilibrium and (**b**) in a dynamical state. The figure introduces various elements and notations used throughout the proceeding sections: a system of coordinates such that the (*x*, *y*) plane coincide with the equilibrium surface of water; the equation of the free surface is encoded in height *ζ*(*x*, *y*; *t*); two additional horizontal planes which are always above/below the free surface; an elementary volume *δV* of horizontal section *δS*.

We consider the liquid to be confined in a tank with flat bottom and with vertical walls but otherwise of arbitrary shape. Vertical pillars of arbitrary sections can be also incorporated. The working assumptions are that the wave amplitudes are small, the liquid is incompressible and the velocity field is irrotational (*i.e*. no vortices are present). A schematic of a rectangularly-shaped water tank is shown in Fig. 1, which will be used to introduce our notation. The density distribution of the fluid at the equilibrium configuration is denoted by $\bar{\rho }$. It is given by:

2 $$\bar{\rho }(x,y,z)=\{\begin{array}{ll}{\rho }_{0} & -h\le z\le \mathrm{0,}\\ 0 & z > 0.\end{array}$$

Throughout, *ρ*_0_ represents the density of water. The density of the fluid in a dynamical configuration is denoted by *ρ*(*x*, *y*, *z*; *t*), which in the notations of Fig. 1 is given by:

3 $$\rho (x,y,z)=\{\begin{array}{ll}{\rho }_{0} & -h\le z\le \zeta (x,\,y;\,t),\\ 0 & z > \zeta (x,\,y;\,t\mathrm{)}.\end{array}$$

The mass current-density is, by definition, the flux of mass per unit area and it can be expressed as ***j*** = *ρ*_0_***v*** in terms of the average velocity of water molecules. It has to obey the continuity equation and the irrotational condition (*i.e*. absence of vortices):

4 $$\frac{\partial \rho }{\partial t}+{\boldsymbol{\nabla }}{\boldsymbol{j}}=0\,\,\& \,\,{\boldsymbol{\nabla }}\times {\boldsymbol{j}}=0.$$

Inside the liquid and away from the surface, *ρ* = *ρ*_0_, hence:

5 $${\boldsymbol{\nabla }}{\boldsymbol{j}}=0\,\,\& \,\,{\boldsymbol{\nabla }}\times {\boldsymbol{j}}=0.$$

The common solution of these two equations can be written as ***j*** = ∇*η*, where *η* is a scalar field satisfying the Laplace equation ∇^2^*η* = 0. At the interface with the fixed walls, the velocity field must be tangent hence the Laplace equation needs to be complemented with the boundary condition ***w*** · ∇*η* = 0 along the fixed walls, where ***w*** is the normal vector to the walls. Since the lateral walls of the container and the pillars are vertical, the elementary solutions of the Laplace equation can be obtain by separation of variables *η*(*x*, *y*, *z*) = *ϕ*(*x*, *y*)*χ*(*z*), which leads to the equation:

6 $$-\frac{1}{\varphi (x,y)}(\frac{\partial }{\partial {x}^{2}}+\frac{\partial }{\partial {y}^{2}})\varphi (x,y)=\frac{1}{\chi }\frac{{\partial }^{2}\chi }{\partial {z}^{2}}={\rm{\Lambda }}\,(=\,{\rm{constant}}\mathrm{)}.$$

In other words, Λ must be among the discrete set of (positive!) eigenvalues ${\{{{\rm{\Lambda }}}_{n}\}}_{n\in {\mathbb{N}}}$ of the 2-dimensional Laplace operator with von Neumann boundary conditions and *ϕ* must be a corresponding (normalized) eigenvector:

7 $$-(\frac{\partial }{\partial {x}^{2}}+\frac{\partial }{\partial {y}^{2}}){\varphi }_{n}(x,y)={{\rm{\Lambda }}}_{n}{\varphi }_{n}(x,y),\quad {\boldsymbol{w}}\cdot {\boldsymbol{\nabla }}{\varphi }_{n}=0.$$

The corresponding *χ* must satisfy the equation:

8 $$\frac{{\partial }^{2}{\chi }_{n}}{\partial {z}^{2}}={{\rm{\Lambda }}}_{n}{{\chi }}_{n},\quad \frac{\partial {{\chi }}_{n}}{\partial z}{|}_{z=-h}=\mathrm{0,}$$

whose unique solution (up to a constant) is:

9 $${\chi }_{n}(z)=A\,\cosh ((z+h)\sqrt{{{\rm{\Lambda }}}_{n}}).$$

At this point, let us define the constants:

10 $${\kappa }_{n}=\frac{1}{{\chi }_{n}}\frac{{\rm{d}}{\chi }_{n}}{{\rm{d}}z}{|}_{z=0}=\sqrt{{{\rm{\Lambda }}}_{n}}\,\tanh (h\sqrt{{{\rm{\Lambda }}}_{n}}),$$

which will play an important role in the following. The conclusion so far is that the scalar field *η* can be always decomposed in elementary modes as:

11 $$\eta (x,y,z;t)=\sum _{n}{A}_{n}(t){\varphi }_{n}(x,y){\chi }_{n}(z\mathrm{)}.$$

The amplitudes *A*_*n*_ play now the role of effective degrees of freedom for the system.

Our next task is to derive the Lagrangian and the equation of motion for these degrees of freedom. Near the moving surface of the liquid, there will be a non-zero density difference $\rho -\bar{\rho }$, and in the limit of small amplitudes, we can define a superficial mass density:

12 $$\sigma (x,y;t)=\mathop{\mathrm{lim}}\limits_{\delta S\to 0}\tfrac{1}{\delta S}{\int }_{\delta V}(\rho (x,y,z;t)-\bar{\rho }(x,y,z)){\rm{d}}V,$$

which can take positive and negative values. Here, *δV* is a volume element centered around the equilibrium surface and whose width is larger than the amplitudes of the waves, hence the free surface of the liquid never intersects the upper and the bottom facets of *δV* (see Fig. 1). If *ζ*(*x*, *y*; *t*) represents the height of the column of fluid at point (*x*, *y*) and moment of time *t*, then:

13 $$\sigma (x,y;t)=\mathop{\mathrm{lim}}\limits_{\delta S\to 0}\frac{1}{\delta S}\int ({\int }_{0}^{\zeta }{\rho }_{0}{\rm{d}}z){\rm{d}}S\,\Rightarrow \,\sigma (x,y;t)={\rho }_{0}\zeta (x,y;t\mathrm{)}.$$

The relation between *σ* and the scalar function *η* can be derived from the continuity equation:

14 $$\frac{{\rm{d}}}{{\rm{d}}t}\int \rho (x,y,z;t){\rm{d}}V+\int {\boldsymbol{j}}(x,y,z;t)\cdot {\rm{d}}{\boldsymbol{S}}=\mathrm{0,}$$

written over volume *δV*. It can be transformed into:

15 $$\frac{{\rm{d}}}{{\rm{d}}t}\frac{1}{\delta S}\int (\rho (x,y,x;t)-\bar{\rho }(x,y,x)){\rm{d}}V+\frac{1}{\delta S}\int {\boldsymbol{j}}(x,y,z;t)\cdot {\rm{d}}{\boldsymbol{S}}=\mathrm{0,}$$

which in the limit *δS* → 0 becomes:

16 $$\frac{\partial \sigma }{\partial t}-{{\boldsymbol{e}}}_{z}\cdot {\boldsymbol{j}}=\mathrm{0,}$$

where we took into account that, in the limit of small wave amplitudes, the width of *δV* is infinitesimally small hence there is no flux of matter through the lateral sides. Also, the upper side of *δV* was chosen in such a way that it doesn’t touches the fluid, hence the only flux of matter is through the bottom facet of Δ*V*. The conclusion is:

17 $$\frac{\partial \sigma }{\partial t}(x,y;t)=\frac{\partial \eta }{\partial z}\,(x,\,y,\,\mathrm{0;}\,t)\,\iff \,\frac{\partial \eta }{\partial z}\,(x,\,y,\,\mathrm{0;}\,t)={\rho }_{0}\frac{\partial \zeta }{\partial t}(x,y;t\mathrm{)}.$$

We now start the construction of the Lagrangian. The kinetic energy of the fluid is:

18 $$\begin{array}{c}T=\tfrac{1}{2}\int {{\boldsymbol{v}}}^{2}{\rm{d}}m=\tfrac{1}{2}\int {{\boldsymbol{v}}}^{2}\rho {\rm{d}}{V}=\frac{1}{2{\rho }_{0}}\int {({\boldsymbol{\nabla }}\eta )}^{2}{\rm{d}}{V}\\ \quad =\frac{1}{2{\rho }_{0}}\int {\boldsymbol{\nabla }}(\eta {\boldsymbol{\nabla }}\eta ){\rm{d}}V=\frac{1}{2{\rho }_{0}}\int \eta {\boldsymbol{\nabla }}\eta \cdot {\rm{d}}{\boldsymbol{S}}\end{array}$$

Note that only the free surface needs to be considered in the above integral because along the fixed walls ∇*η* · d***S*** = 0. Hence:

19 $$T=\frac{1}{2{\rho }_{0}}\int \eta (x,y,\,\mathrm{0;}\,t)\frac{\partial \eta }{\partial z}(x,y\,\mathrm{,0;}\,t){\rm{d}}S.$$

The gravitational potential energy of the fluid, relative to its equilibrium configuration, is:

20 $$U=\int gz\,{\rm{d}}m-\int gz{\rm{d}}\bar{m}=\int (\rho -\bar{\rho })gz{\rm{d}}V={\rho }_{0}g\int ({\int }_{0}^{\zeta }z{\rm{d}}z){\rm{d}}S,$$

or:

21 $$U=\tfrac{1}{2}{\rho }_{0}g\int \zeta {(x,y;t)}^{2}{\rm{d}}S.$$

The conclusion at this step is that the Lagrangian *L* = *T* − *U* of the full body of fluid can be written entirely in terms of a surface integrals:

22 $$L=\frac{1}{2{\rho }_{0}}\int \eta (x,y,\mathrm{0;}\,t)\frac{\partial \eta }{\partial z}(x,y,\mathrm{0;}\,t){\rm{d}}S-\tfrac{1}{2}{\rho }_{0}g\int \zeta {(x,y;t)}^{2}{\rm{d}}S,$$

or

23 $$L=\tfrac{1}{2}\int \eta (x,y,\mathrm{0;}\,t)\frac{\partial \zeta }{\partial t}(x,y;t){\rm{d}}S-\tfrac{1}{2}{\rho }_{0}\,g\int \zeta {(x,y;t)}^{2}{\rm{d}}S.$$

The set of solutions ${\{{\varphi }_{n}\}}_{n\in {\mathbb{N}}}$ is a complete orthonormal set of functions, hence we can expand:

24 $$\zeta (x,y;t)=\sum _{n}{C}_{n}(t){\varphi }_{n}(x,y\mathrm{)}.$$

Due to relation (17), the coefficients *A*_*n*_ in (11) and *C*_*n*_ from above are related as:

25 $${A}_{n}(t)\frac{\partial {\chi }_{n}}{\partial z}{|}_{z=0}={\rho }_{0}\,{\dot{C}}_{n}(t),\quad ({\dot{C}}_{n}=\frac{{\rm{d}}{C}_{n}}{{\rm{d}}t}),$$

hence:

26 $$\eta (x,y,\mathrm{0;}\,t)={\rho }_{0}\sum _{n}\frac{1}{{\kappa }_{n}}{\dot{C}}_{n}(t){\varphi }_{n}(x,y\mathrm{)}.$$

Plugging these expansions into (23) and using the orthonormality of *ϕ*_*n*_’s:

27 $$L=\tfrac{1}{2}\sum _{n}\frac{{\rho }_{0}}{{\kappa }_{n}}({\dot{C}}_{n}{(t)}^{2}-g{\kappa }_{n}\,{C}_{n}{(t)}^{2}).$$

The Euler-Lagrange equations of motions are:

28 $${\ddot{C}}_{n}(t)=-g{\kappa }_{n}{C}_{n}(t),$$

leading to the frequency of the resonant modes:

29 $${f}_{n}=\tfrac{1}{2\pi }\sqrt{g\sqrt{{{\rm{\Lambda }}}_{n}}\,\tanh (h\sqrt{{{\rm{\Lambda }}}_{n}})}.$$

The resonant standing wave pattern corresponding to this frequency is *ϕ*_*n*_, defined by the eigenvalue equation (7).

#### Example: The rectangular tank

We consider the case of a rectangular tank of length *L* and width *W*. In this case the solutions of (7) are given by:

30 $${{\boldsymbol{\varphi }}}_{{\boldsymbol{k}}}(x,y)=A\,\cos ({k}_{x}\,x)\,\cos ({k}_{y}\,y),\quad {{\rm{\Lambda }}}_{{\boldsymbol{k}}}={k}^{2}={k}_{x}^{2}+{k}_{y}^{2},$$

where ***k*** = (*k*_*x*_, *k*_*y*_) is a two component vector which takes the quantized values:

31 $${\boldsymbol{k}}=(\frac{\pi n}{L},\frac{\pi m}{W}),\quad n,m\in {\mathbb{N}}.$$

The corresponding frequencies are:

32 $${f}_{{\boldsymbol{k}}}=\tfrac{1}{2\pi }\sqrt{gk\,\tanh (kh)}.$$

In the infinite volume limit *L*, *W* → ∞ and for *kh* << 1, $\tanh (kh)\approx kh$ and the pulsation of the modes become ${{\boldsymbol{\omega }}}_{{\boldsymbol{k}}}=\sqrt{gh}k$. This is a linear dispersion law which gives a constant speed of waves:

33 $$c=\frac{{\rm{d}}{\omega }_{{\boldsymbol{k}}}}{{\rm{d}}k}=\sqrt{gh},\quad ({\rm{shallow}}\,{\rm{water}}\,{\rm{limit}}),$$

in full agreement with the shallow water theory [^14^, p. 27]. Furthermore, in the limit *h* → ∞, $\tanh (kh)\approx 1$) and the pulsation of the modes becomes ${{\boldsymbol{\omega }}}_{{\boldsymbol{k}}}=\sqrt{gk}$, hence a non-linear dispersion law, leading to an anomalous wavelength:

34 $$\lambda =\frac{2\pi }{k}=\frac{g}{2\pi {f}^{2}},\quad ({\rm{deep}}\,{\rm{water}}\,{\rm{limit}}\,),$$

in full agreement with the deep water theory [^14^, p. 39]. For a narrow channel *W* << *L* along the *x*-axis, the lower part of the spectrum is characterized by:

35 $${\varphi }_{k}(x)=\sqrt{\frac{2}{L}}\,\cos (k\,x),\quad {f}_{k}=\tfrac{1}{2\pi }\sqrt{gk\,\tanh (kh)},\quad k=\frac{2n\pi }{L},\quad n\in {\mathbb{N}}.$$

## Experimental Investigations

### Results: Dispersion for narrow wave-channels

**Figure 2:**
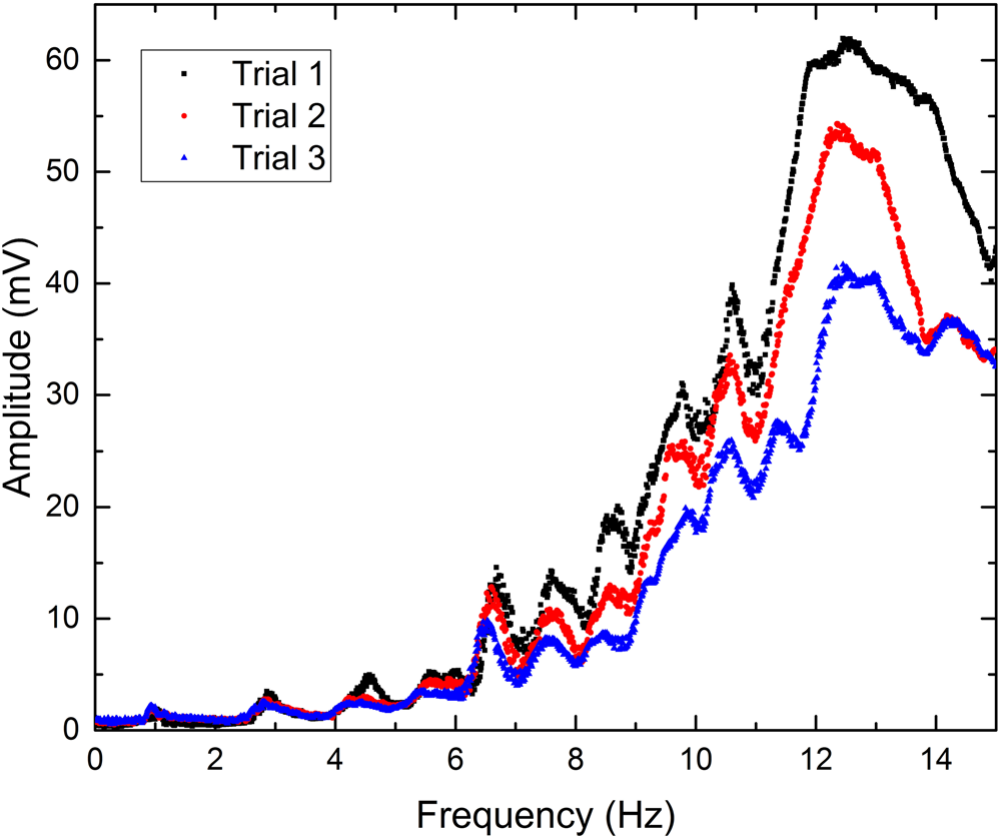
Quantitative data acquired for a 19.75 cm long flat-walled channel filled with 1.4 cm H2O demonstrating repeatability. Slight variations in resonant frequencies are observable, but can be explained by variations in water height within the channel.

**Figure 3:**
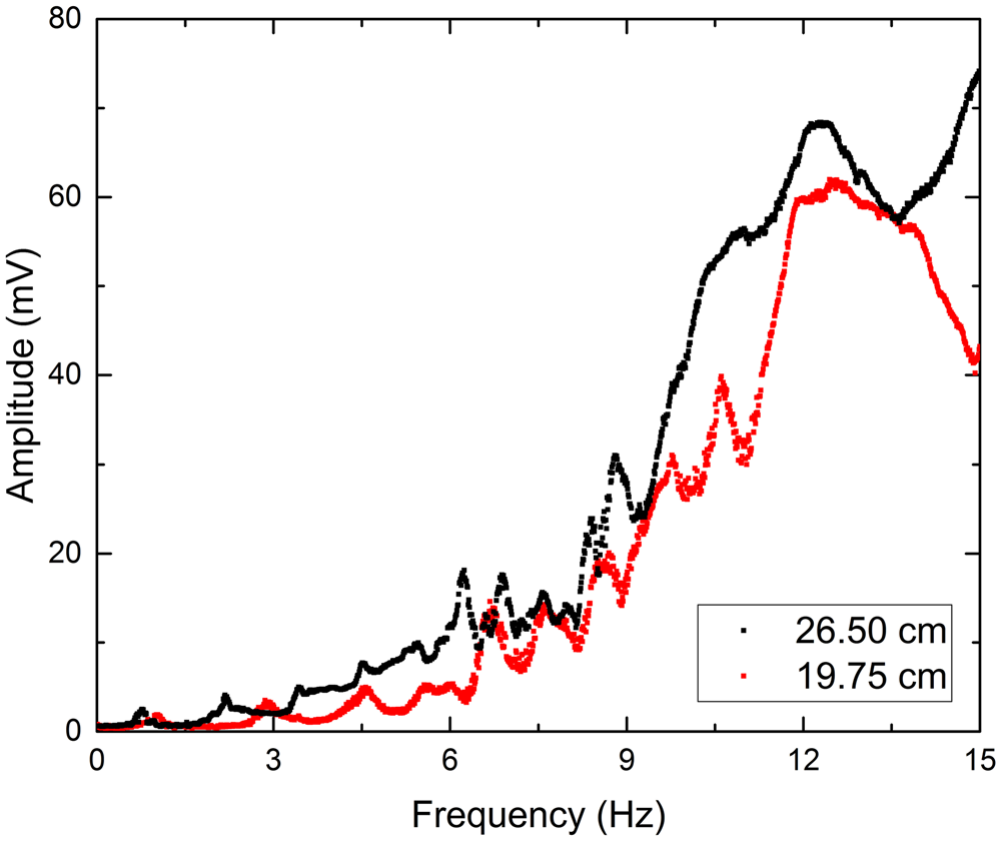
Experimental data comparing measured signal of transmitted light of flat-walled channels with lengths, L = 19.75 cm and L = 26.5 cm.

The experiments for flat-walled channels utilized a channel of width, *W* = 2 cm and two varying lengths (*L* = 19.75 cm and *L* = 26.5 cm). Utilizing the 650 nm laser diodes positioned incident to the water allowed the user to acquire quantitative data pertaining to the amplitude of the resonant modes. Channels were filled with 1.4 cm of water and actuated between frequencies of 0.01 and 15 Hz in 0.01 Hz steps. The RMS signal for a 19.75 cm long flat-walled channel over three separate experiments is shown in Fig. 2. A comparison of the signals for the two different length flat-walled channels is shown in Fig. 3. As shown, the resonant frequencies change according to Equation (32).

In order to verify that the frequencies at which peaks appear in the acquired signal are resonant modes, they were manually inputted into the function generator to actuate the system. It was then possible to observe (either via imaging to a screen or directly viewing the water) that these frequencies were indeed resonant modes.

**Figure 4:**
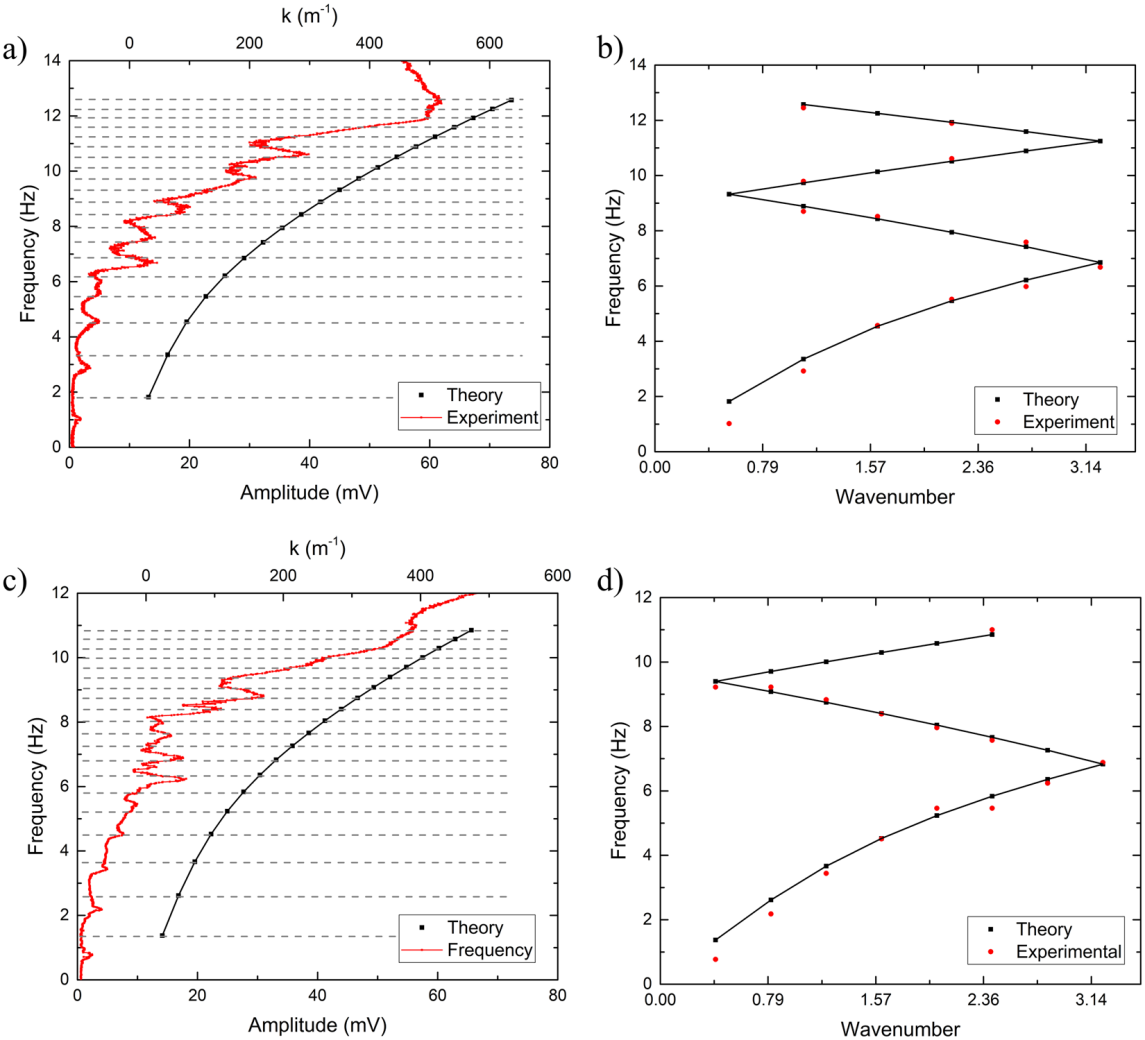
(**a**) Data comparing theoretical and experimental resonant frequencies for a 19.75 cm long, 2 cm wide, flat-walled channel filled with 1.4 cm high water. Black squares correspond to theory and red dots correspond to experimental data. Theory points correspond to upper x-axis, while experimental points correspond to lower x-axis. (**b**) Comparison of the phonon spectrum for 19.75 cm long flat-walled channel. (**c**) Data comparing theoretical and experimental resonant frequencies for a 26.5 cm long, 2 cm wide, flat-walled channel filled with 1.4 cm high water. Black squares correspond to theory and red dots correspond to experimental data. Theory points correspond to upper x-axis, while experimental points correspond to lower x-axis. (**d**) Comparison of the phonon spectrum for a 26.5 cm long flat-walled channel.

A comparison between the theory and experiment for the two channels is shown in Fig. 4. The data in Fig. 4a was acquired using a channel of length, *L* = 19.75 cm, and height of water, *h* = 1.4 cm. The black, square data points correspond to the theory and the red, circular points correspond to the experiment. The experimental points correspond to the lower x-axis only, while the theoretical points correspond solely to the upper x-axis. Both share a common y-axis. As shown, at frequencies where resonant modes exist, a signal with higher intensity is acquired by the photodiode. Figure 4b demonstrates a typical phonon spectrum for water waves in a flat-walled waveguide. In anticipation of the periodic modulations introduced next, the data is shown as function of the wavenumber *k* · *a*, with *a* the periodicity of the modulations. The wavenumber axis is folded into the interval [−*π*, *π*] but only the positive part is shown. Figure 4c demonstrates the theory and experimental data for a flat-walled channel of *L* = 26.5 cm, and height of water, *h* = 1.4 cm. The black, square points correspond to the theory and the upper-x axis only, while the red, circular points show the experimental data and correspond to the lower x-axis. The phonon spectrum for the longer channel is demonstrated in Fig. 4d. The spectra for both lengths are missing some experimental data points, particularly at higher frequencies. This can be explained as a consequence of the measuring system’s resolution. Due to the length limitations of the waveguide, higher resonant modes are difficult to distinguish because the distance between nodes decreases. As this occurs, it becomes harder for the laser (with a set spot size) to distinguish a clear resonance.

### Periodically patterned wave-channel

**Figure 5:**
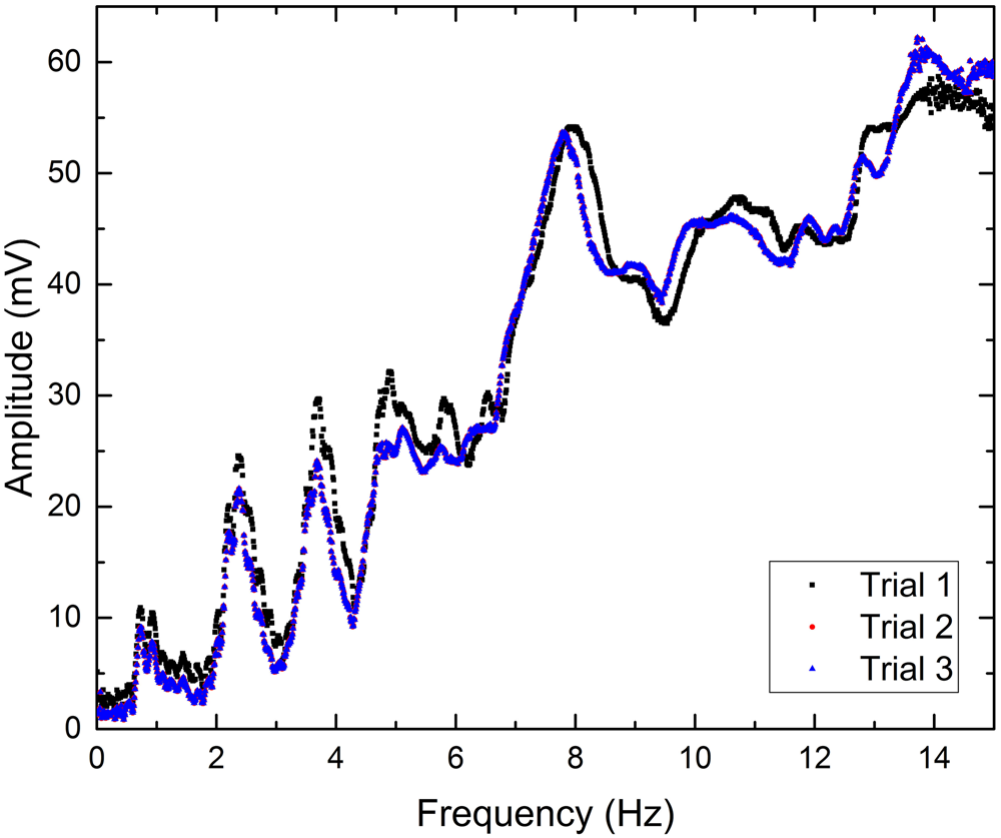
Data demonstrating the repeatability of the measurement for a 26.5 cm long periodic channel.

**Figure 6:**
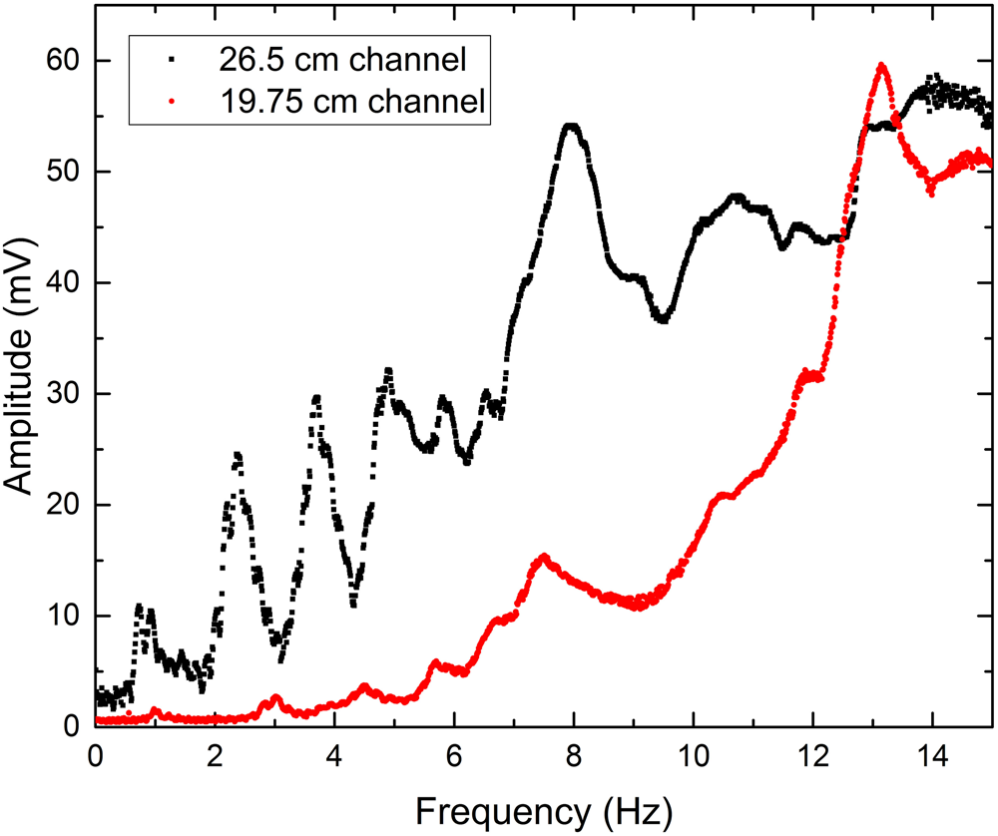
Comparison of 2 different lengths of periodic channels.

A periodically patterned water wave guide was designed by 3D printing a channel with teeth spaced 1.12 cm apart. In order to study the properties of water within the wave guide, two channels of length, *L* = 19.75 cm and *L* = 26.5 cm were fabricated. The channels were filled to a height of water, *h* = 1.4 cm and excited over frequencies of 0.01 Hz and 15 Hz with 0.01 Hz step sizes. The measured signals for a 26.5 cm long periodic channel over three separate trials are shown in Fig. 5. Slight shifts in resonant frequencies are visible between the trials. A comparison of periodically patterned channels of two lengths is shown in Fig. 6. The red circle points correspond to a channel with, *L* = 19.75 cm and the black square points to *L* = 26.5 cm.

**Figure 7:**
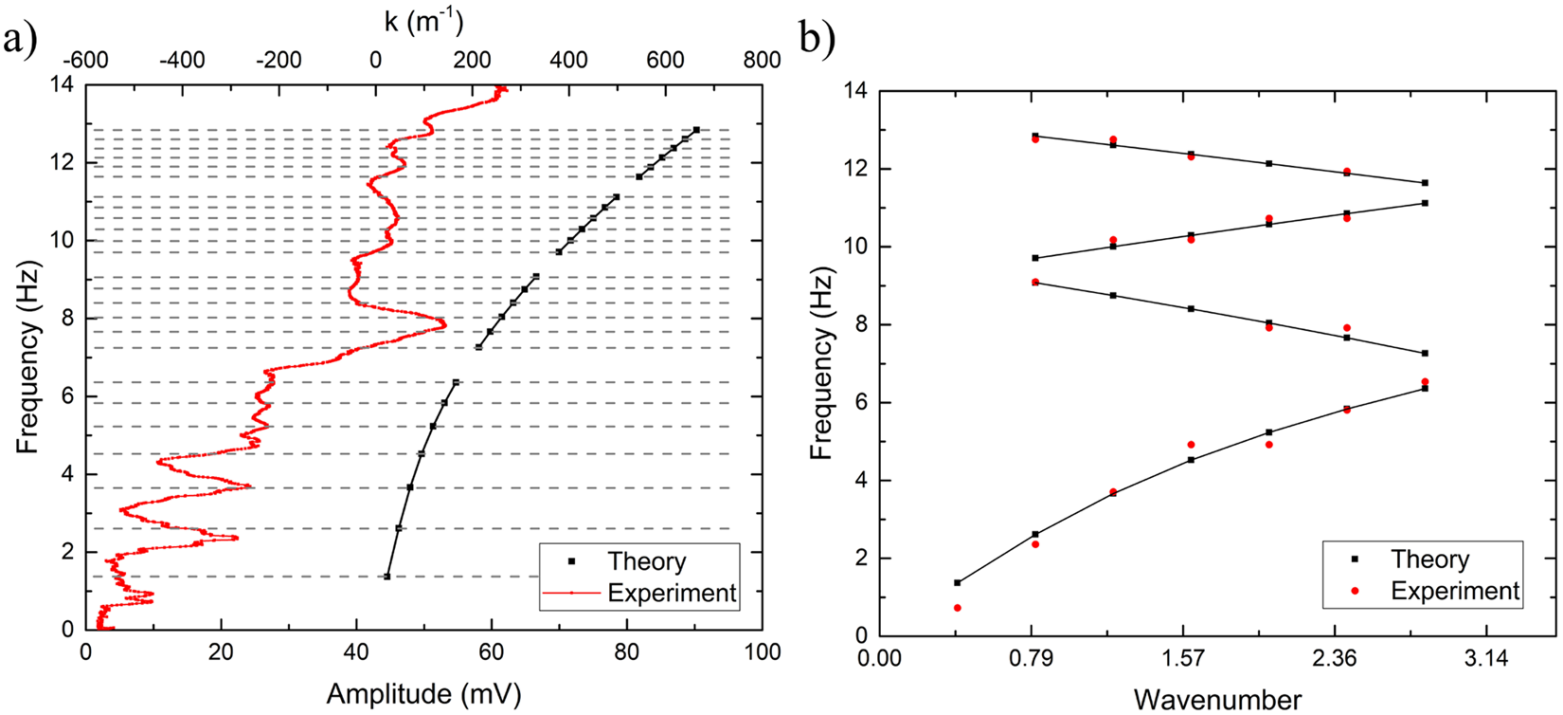
Data for a 26.5 cm long, periodic channel filled with 1.4 cm high water. Black squares correspond to theory and red dots correspond to experimental data. (**a**) Comparison of theory and experimental resonant frequencies. Theory points correspond to upper x-axis, while experimental points correspond to lower x-axis. (**b**) Comparison of the phonon spectrum for a periodic channel.

A comparison of the experimental resonant frequencies of a 26.5 cm long periodically patterned channel and theoretical values (Eq. (35)) is show in Fig. 7. When the water is in resonance, the amplitude of the signal is greater and a peak appears. This behavior is similar to that of the flat-walled channel experiments. The experimental data corresponds to the lower x-axis and y-axis, while the theoretical points correspond to the upper x-axis and y-axis. Gray dashed lines have been added to demonstrate where expected resonances should occur. Figure 7b shows the phonon spectrum of the periodic channel with the wavenumber axis folded as previously explained.

## Discussion

In all reported experiments, the resonant modes are dependent on the height of the water within the channel as governed by Eq. (32). Maintaining the height of the water is extremely important. Due to the small step size in frequency (0.01 Hz), the experiments took approximately 5 hours to perform. Throughout the duration of that time, it is possible that some of the water in the channel evaporated. As a result, it is possible that a measurable shift in resonant frequencies occurred. However, it is believed that throughout the course of the reported experiment significant evaporation did not occur. The repeatability of the experiment for a 19.75 cm long flat-walled channel is show in Fig. 2. The data suggests that the experiment is repeatable with small deviations between trials. This behavior can be accounted for by considering the inaccuracy in the height of the water in the waveguide. Since the channels are filled by volume, if slightly off, the resonant frequencies will be different. In addition, if any evaporation occurs, shifts in resonant frequencies are possible. Also, the signal is highly dependent on the initial intensity of the transmitted light; therefore, if any change in it occurs, the overall amplitude varies. At higher resonant modes (>7), the intensity of the transmitted signal is greater. This can be explained by the Beer-Lambert law, which states that as the height of the fluid in the channel decreases, the intensity of the transmitted signal increases. Thus, as higher resonant modes form and the peaks for the waves are not as high as at lower ones, the height of the fluid, is smaller. Thus, the intensity of the transmitted signal is larger and an increase is reflected in the acquired data.

Similarly, Fig. 2 demonstrates the repeatability of a 26.5 cm longer periodically patterned waveguide. Once again, the data suggests the experiment is repeatable with small deviations between trials. The same reasons the variability exists in the flat-walled channels can be applied to the periodic channels. A comparison of two different lengths of periodic waveguides is shown in Fig. 6. As in the case of the flat-walled channel, the signal is clearly different between the two lengths. A difference in signal is expected since length is one of the main factors that governs the resonant frequencies of the water within the channels. For periodic systems, spectral gaps are expected. It is difficult to identify the location of said gaps solely from the RMS signal, but they can be seen when plotted against the theoretical values.

The comparison of experimental and theoretical data for a flat-walled channel shown in Fig. 4 is a representative plot that demonstrates how the theory aligns with the experiment for two different lengths. Raw data compared with theory is show in (a) and (c). On the right, (b) and (d) demonstrate the spectrum where the x-axis corresponds to the situation when the k vector is modified. For a flat-walled channel, we assume no gaps open and resonant frequencies are present in the system. However, for a periodically patterned channel, gaps should open up where the spectrum winds back in the zig-zag fashion. A comparison of the experimental and theoretical data for a periodic channel is show in Fig. 7. The data suggests that a resonance is not measured at every theoretical point, particularly those where the spectrum winds back on itself. This behavior can be explained by the presence of gaps due to the periodic spacing of the teeth.

## Methods

### The experimental apparatus

**Figure 8:**
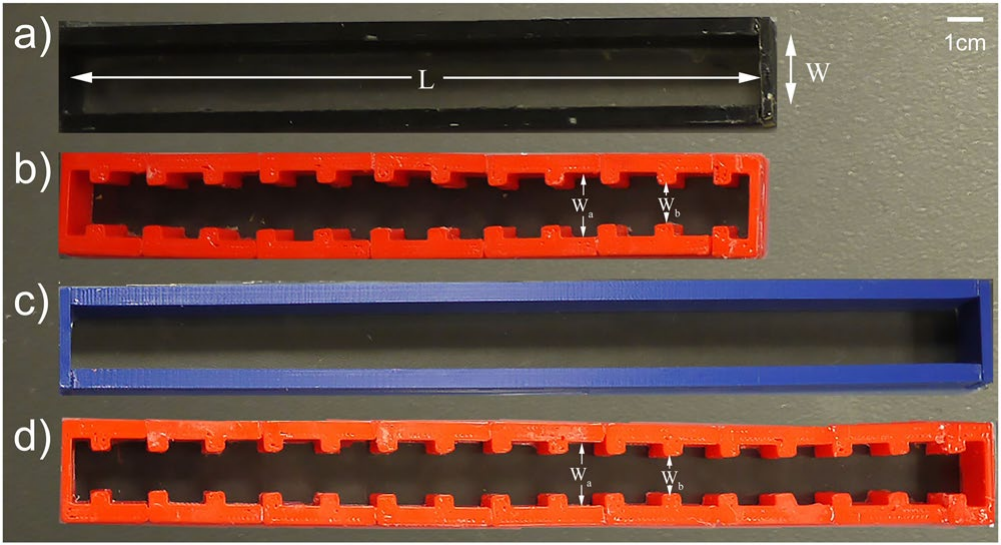
Various 3D printed channels utilized for experimental testing. (**a**) Flat-walled channel, *L* = 19.75 cm, *W* = 2 cm. (**b**) Periodic channel, *L* = 19.75 cm, *W*_*a*_ = 2 cm, *W*_*b*_ = 1.2 cm. (**c**) Flat-walled channel, *L* = 26.5 cm, *W* = 2 cm. (**d**) Periodic channel, *L* = 26.5 cm, *W*_*a*_ = 2 cm, *W*_*b*_ = 1.2 cm.

**Figure 9:**
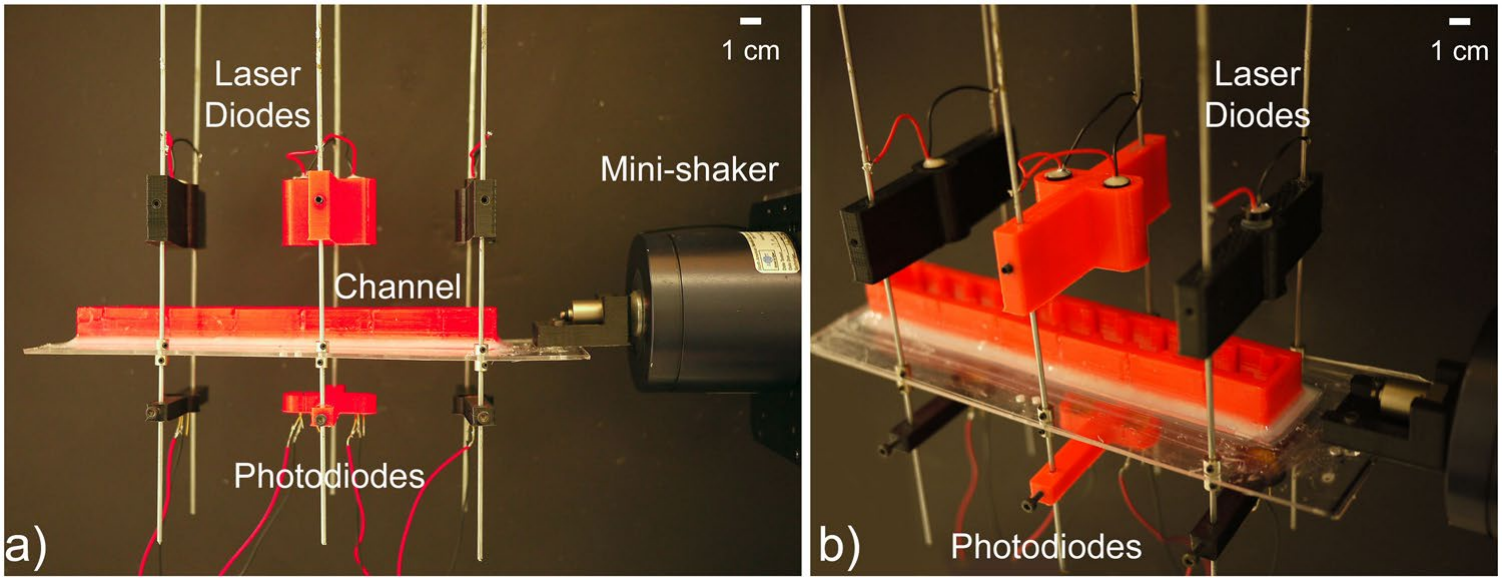
Image of the experimental apparatus. The mini-shaker drives the system while the laser/photodiode pairs are the sensing mechanism. (**a**) Side-view of the experimental apparatus (function generator, power amplifier, laser power supply, and USB GPIB box not shown). The red and black wires on the lasers attach to the rods, which act as conduits to bring power to the laser diodes. The red and black wires attached the the photodiodes lead to the GPIB box and ultimately the computer for data collection. (**b**) Angled view of the apparatus to demonstrate how wires are attached and show a *L* = 19.75 cm periodic channel attached to the system. The acrylic/channel can be swapped out depending on the geometry of choice.

Flat-walled and periodic channels were 3D printed using acrylonitrile butadiene styrene (ABS) shown in Fig. 8. At frequencies much higher than those actuated in the present experiments, harder materials should be used for the walls. The waveguides were positioned atop an acrylic sheet suspended by 20 cm long pendulum rods to maintain small oscillations (that is, *θ* < 5°). In order to prevent leakage of fluid, a seal was created using silicone caulk between the channel and the acrylic. Adafruit Industries 5 mW 650 nm laser diodes were positioned above the channel and permitted to emit light through the water and acrylic onto an Opto Diode Corp 9.91 mm × 4.28 mm photodiode positioned underneath. The experimental apparatus can be seen in Fig. 9 (the channel can be interchanged depending on geometry of interest). The system was actuated by using an HP 33120A function generator connected to a Brüel & Kjær Power Amplifier Type 2718 which drove a Brüel & Kjær 4810 Mini-shaker. The actuator was attached to the acrylic sheet using two bolts. The function generator was computer-controlled by a previously reported^20^ custom LabVIEW program via a USB GPIB cable and a signal was transmitted to scan over frequencies of 0.01 Hz to 15 Hz in 0.01 Hz steps. The actuator excited the system in one direction.

This system made it possible to take a measurement of the intensity of transmitted light utilizing laser/photodiode pairs, which identified resonant frequencies of the fluid within the channel. As the water approached a resonant frequency, the intensity of the transmitted light increased. This behavior occurs because when the fluid is off-resonance, the turbulent nature of the water refracts the light away from the photodiode, and decreases the intensity of the attenuated light. As the system becomes resonant, the light is allowed to focus on the photodiode and the highest intensity of the light is transmitted. The intensity of the transmitted signal is measured by the Beer-Lambert Law^21^:

36 $$\frac{I}{{I}_{0}}={e}^{-\alpha h},$$

Where *I* is the intensity of the signal, *I*_0_ is the intensity of the signal with no fluid present, *α* is the absorption coefficient, and *h* is the height of the fluid in the channel. Therefore, as the height of the water in the channel changes, the intensity of the signal varies. Recalling Eq. (32), it is clear that the resonant modes also depend on the height of the water in the channel. The experiments reported in this paper were performed with a height of water of *h* = 1.4 cm.

#### Previous methods

Prior to finalizing the above-mentioned suspended design of the actuating system, various methods were tested and deemed inadequate for testing. The first design utilized a circular tank of water which rested on a raised sheet of Poly(methyl methacrylate) (PMMA). The fluid was actuated by a cylindrical pin positioned off-center. In this configuration, waves propagated quite well at higher frequencies (>20 Hz), but poorly at lower ones. Resonant modes were determined by observation of the water within the system and images could be acquired on a screen positioned underneath the raised PMMA sheet. Standing waves formed at resonant modes and were relatively easy to observe. While this approach worked quite well with higher frequencies, below 20 Hz it was extremely difficult to distinguish resonant modes by eye.

In order to measure the resonant modes at lower frequencies, the same apparatus was utilized, but a laser was positioned at a 45° angle such that the light hit the water at the center of the circular tank. A detector was placed opposite it to detect the reflected light from the surface of the water. Due to the multiple-interface system, it was extremely difficult to isolate the reflected laser light from the surface of the water onto the detector. The data was inconclusive at low frequencies and a new method was pursued. In addition, the circular tanks were replaced with flat-walled rectangular ones of varying lengths.

The second design used a glass tank of water 2.54 cm tall in which the rectangular channels were positioned. The fluid was actuated utilizing the same cylindrical pin as previously described. For the flat-walled circular tanks this method of actuating the fluid proved quite successful. However, upon switching to non-circular tanks, the waves did not propagate throughout the system as expected due to an inefficient amount of energy required to excite the wave across the full length of the channel. It was hypothesized that switching the small-tipped cylinder with a wider paddle would produce enough energy to excite the system. A tip was still desired, so the geometry was changed by angling the walls varying degrees. Paddles of various sizes were used, but proved inefficient at exciting the water. It was found that a paddle with angled walls of 45° could sufficiently excite the water across the channel length. This paddle was capable of exciting the water throughout the system for flat-walled rectangular tanks; however, could not do so for channels with periodicity. Therefore, a new method of exciting the water was pursued.

**Figure 10:**
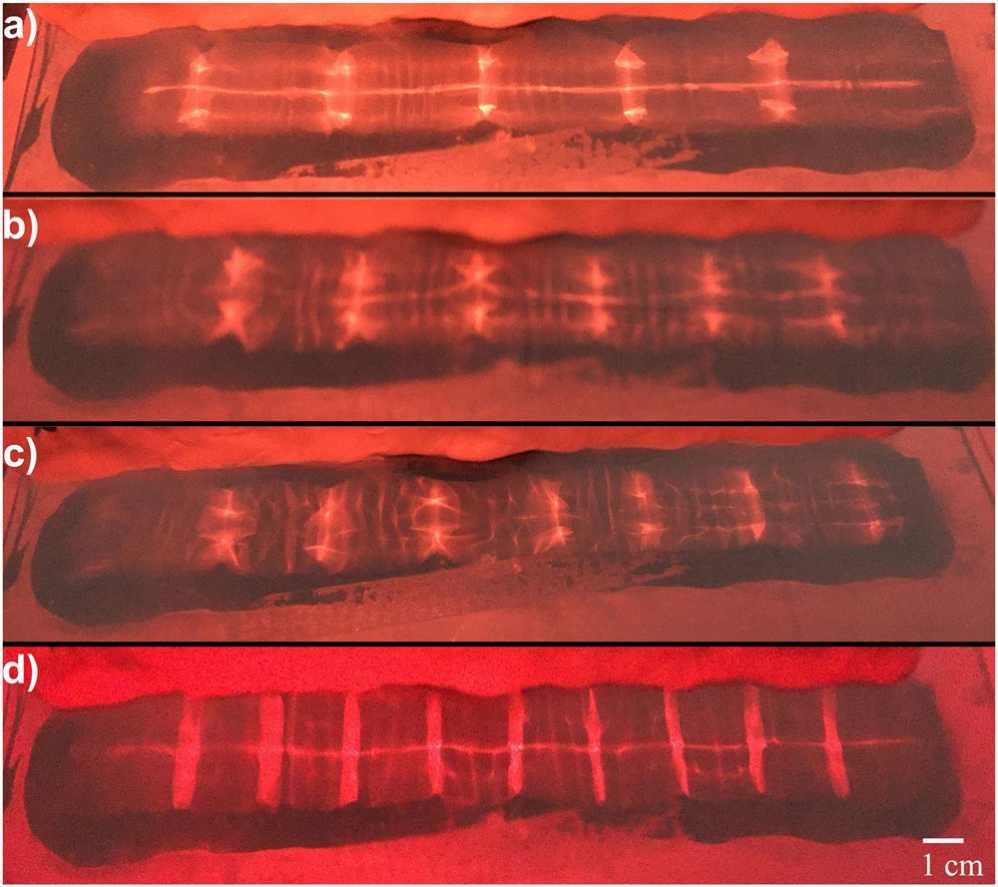
Resonant modes in a 19.75 cm flat-walled channel demonstrated by bright lines between channel walls. (**a**) 5 resonant modes with *λ* = 5.7 cm, (**b**) 6 resonant modes with *λ* = 5.0 cm, (**c**) 7 resonant modes with *λ* = 4.5 cm, and (**d**) 9 resonant modes with *λ* = 3.1 cm.

The next design utilized a glass sheet atop an actuator, similar to a Chladni plate. The channels were placed and sealed on the plate for excitation. A stroboscope was designed using a red LED that flashed at the same frequency of the excitation. A switch was incorporated to allow the user to select the strobe light or steady light for imaging. This method of excitation worked rather well, and resonant modes at low frequencies were acquired (see Fig. 10).

While this method of water excitation proved quite successful, it was not without flaw. Determination of the resonant modes solely relied on observation of the modes in the channel. Videos and images were acquired demonstrating these observations. For flat-walled channels, this method seemed adequate. Once a periodic channel was installed, this method no longer was viable since resonant modes were not as clearly defined. It became apparent that observing resonant modes in channels with periodicity would not be possible. In order to determine what was happening in said channels, the laser/photodiode apparatus described earlier was adapted.

## Conclusions

This paper presents a unifying theory for shallow and deep water wave behavior which provides a mathematical way to predict resonant modes in various fluid waveguides. In addition, we have provided an experimental platform capable of acquiring data demonstrating the phononic spectra for flat-walled and periodically-patterned channels. Actuating a suspended system and utilizing the laser/photodiode pairs, it was shown that spectral gaps are absent for flat-walled channels, but exist in the periodically-patterned ones. This unifying theory and experimental platform provides a flexible system that is useful for future studies of various waveguides.
